# Studies on the interactions of retinal dopamine with choroidal thickness in the chicken

**DOI:** 10.1007/s00417-022-05837-w

**Published:** 2022-10-04

**Authors:** Ute Mathis, Marita Feldkaemper, Hong Liu, Frank Schaeffel

**Affiliations:** 1 Ophthalmic Research Institute, University of Tuebingen, Tuebingen, Germany; 2 Institute for Molecular and Clinical Ophthalmology Basel (IOB), Basel, Switzerland; 3 Zeiss Vision Lab, Ophthalmic Research Institute, University of Tuebingen, Tuebingen, Germany

**Keywords:** Myopia, Choroid, Atropine, Dopamine, Chicken

## Abstract

**Purpose:**

Recently, an increasing number of studies relied on the assumption that visually induced changes in choroidal thickness can serve as a proxy to predict future axial eye growth. The retinal signals controlling choroidal thickness are, however, not well defined. We have studied the potential roles of dopamine, released from the retina, in the choroidal response in the chicken.

**Methods:**

Changes in retinal dopamine release and choroidal thickness changes were induced by intravitreal injections of either atropine (250 µg or 360 nMol), atropine combined with a dopamine antagonist, spiperone (500 µMol), or spiperone alone and were tracked by optical coherence tomography (OCT). To visually stimulate dopamine release, other chicks were exposed to flicker light of 1, 10, or 400 Hz (duty cycle 0.2) and choroidal thickness was tracked. In all experiments, dopamine and 3,4-Dihydroxyphenylacetic acid (DOPAC) were measured in vitreous, retina, and choroid by high-performance liquid chromatography with electrochemical detection (HLPC-ED). The distribution of the rate-limiting enzyme of dopamine synthesis, tyrosine hydroxylase (TH), neuronal nitric oxide synthase (nNOS), vascular endothelial growth factor (VEGF), and alpha2A adrenoreceptors (alpha2A-ADR) was studied in the choroid by immunofluorescence.

**Results:**

The choroid thickened strongly in atropine-injected eyes, less so in atropine + spiperone–injected eyes and became thinner over the day in spiperone alone-, vehicle-, or non-injected eyes. Flickering light at 20 lx, both 1 and 10 Hz, prevented diurnal choroidal thinning, compared to 400 Hz, and stimulated retinal dopamine release. Correlation analysis showed that the higher retinal dopamine levels or release, the thicker became the choroid. TH-, nNOS-, VEGF-, and alpha2A adrenoreceptor–positive nerve fibers were localized in the choroid around lacunae and in the walls of blood vessels with colocalization of TH and nNOS, and TH and VEGF.

**Conclusions:**

Retinal DOPAC and dopamine levels were positively correlated with choroidal thickness. TH-positive nerve fibers in the choroid were closely associated with peptides known to play a role in myopia development. Findings are in line with the hypothesis that dopamine is related to retinal signals controlling choroidal thickness. 
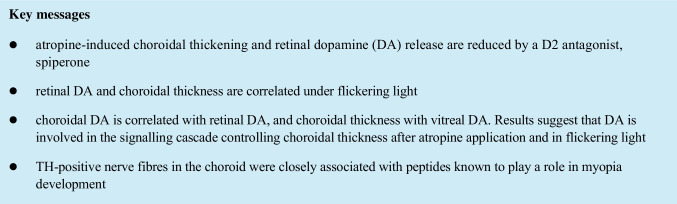

## Introduction

To evaluate the potency of interventions to reduce the progession of myopia, axial eye growth needs to be compared in untreated and treated animal models, or children [1]. Unfortunately, statistically confirmed results require extended observation periods (months or years). To obtain information at shorter time frames as to whether a treatment is promising, an increasing number of studies relied on the assumption that visually induced changes in choroidal thickness can predict future axial eye growth (i.e., [2–6]; reviews [7–9]). For instance, atropine eye drops were found to induce choroidal thickening which was assumed to predict their long-term inhibitory effect on myopia. Based on their large multi-dose atropine study in children in Hong Kong (the “LAMP” study), Yam et al. [10] concluded that “Low concentration atropine induced a choroidal thickening effect along a concentration-dependent response throughout the treatment period. The choroidal thickening was associated with a slower SE (spherical equivalent) progression and AL (axial) elongation among all the treatment groups. Choroidal response can be used for assessment of long-term treatment outcomes ….” Also in chickens, atropine caused prominent choroidal thickening although the changes were transient and lasted only a few hours [11]. It is clear that a better understanding of the retinal signals that alter choroidal thickness is needed. The retinal pigment epithelium (RPE) plays an important role since it relays the retinal output to the choroid and the sclera [12]. As first proposed by Nickla et al. [13], one of the biochemical signals that the retina may use to control choroid thickness could be dopamine [14–18]. All five dopamine receptors are expressed on the RPE, both on the apical side and the basolateral side [19]. Retinal dopamine release is suppressed in the chicken when the retinal image has low contrast or low luminance [20] and both conditions stimulate myopia. Intravitreal atropine injections exert a profound but transient stimulatory effect on retinal dopamine release at a dose of 250 µg [16]. At this dose, atropine also blocked development of deprivation myopia completely [21]. An increase in dopamine release from the retina at the same dose of atropine was found by Thomson et al. [22] but no changes were observed at lower doses (10 µg and 0.1 µg).

Since the relationship between retinal dopamine release and choroidal thickness appears complex and does not always follow a simple prediction (more dopamine – > choroid thicker), we have performed three further experiments: (1) We tested whether a dopamine antagonist, spiperone, can reduce choroidal thickening, induced by atropine. (2) We tested whether changes in dopamine release, induced by flickering light or by brighter office light, are related to changes in choroidal thickness. In both experiments, we measured the corresponding dopamine and DOPAC levels in the vitreous and retina and, in particular, in the choroid. (3) We performed an immunohistochemical analysis of the distribution of tyrosine hydroxylase (TH), the rate-limiting enzyme in dopamine synthesis, in the choroid and studied colocalizations with peptides known to be involved in the visual control of eye growth.

## Material and methods

### Animals

One-day-old male White Leghorn chickens were obtained from a local hatchery (Kilchberg, Germany). They were raised under an 11/13 h light/dark cycle, with the light phase starting at 8:00 a.m. Water and food were supplied ad libitum. Illumination was provided by light bulbs that produced an ambient illuminance of about 500 lx on the cage floor (measured with a calibrated photocell [United DetectorTechnology, USA] in photometric mode). Animals were studied between post-hatch ages P14 to P21. In total, 70 chickens were studied. All experiments were conducted in accordance with the ARVO statement for the use of animals in ophthalmic and vision research and were approved by the University Committee of Tuebingen for experiments involving animals (AK 01/19G).

### Intravitreal injections

Intravitreal injections were performed using a 0.5-mL insulin syringe (30-gauge needle, BD Medical, Le Pont deClaix, France). They consisted of atropine sulfate monohydrate (250 µg/360 nMol, > 97%, Sigma-Aldrich, Deisenhofen, Germany) in 25 µL saline, or spiperone (500 µMol, Sigma-Aldrich, Deisenhofen, Germany) in 25 µL ascorbic acid (1 mg/mL, pH 7.4), or a combination of both atropine and spiperone in 25 µL ascorbic acid, while the fellow eye received 25 µL saline or 25 µL ascorbic acid, respectively. Alternatively, both eyes received 25 µL saline. The atropine dose was selected based on previous experiments [21], where daily injections completely blocked myopia induced by wearing − 7D lenses. The dose of spiperone was also selected based on previous experiments. Thomson et al. [17] had shown that daily injections of 500 µMol of spiperone suppressed the protective effects of dopamine or a dopamine agonist against the development of both deprivation myopia and lens-induced myopia. Ashby and Schaeffel [23] had found that 500 µMol spiperone blocked the protective effect of bright light on deprivation myopia in chickens. All injections were performed under mild ether anesthesia. The intravitreal injection procedure in the chicken is well established (e.g., [11, 24–26]) and, as in previous studies, we never observed ocular inflammations.

### Measurements of choroidal thickness in alert chickens

We had found earlier [27–30] that OCT represents a fast and convenient technique to measure choroidal thickness in unanesthetized, alert chickens. Chicks were held by hand in front of an OCT (HRA + OCT Spectralis, SN 10,543, 04/2016, Heidelberg Engineering; resolution mode: high speed, scan angle: 30°, scan type: B-scan, 768 × 496 pixels, line scan, eye tracking not engaged, scan rate: of the live image 8.8 frames/s, and measurements at 1060 nm). For the measurements, the position of the chicken’s head was manually aligned until the cornea was aligned about perpendicular to the axis of the OCT camera and the scan of the fundal layers became visible on the screen. Optimal alignment of the eye was assumed when the light patch representing the pupil in the left window on the screen was centered and a high contrast and horizontally aligned scan of the fundal layers became visible in the window on the right (a screenshot can be found in Mathis et al. [28]). Five to ten OCT scans were stored for each eye and time point; because chickens do not maintain stable fixation, only 3–5 were selected for analysis. Choroidal thickness was manually measured as the distance from the retinal pigment epithelium to the outer boundary of the choroid [29], using the publicly available software ImageJ (https://imagej.nih.gov/ij/). An interobserver correlation analysis for the OCT measurements of choroidal thickness was performed in a previous study in which both observers were blinded with regard to the treatment of the chickens [29] and showed a correlation coefficient of R = 0.937. In the current study, standard deviations from repeated measurements in the same eyes ranged from 4 to 10 µm, considerably smaller than the interindividual variability among different eyes (between 20 and 50 µm). Choroidal thickness was measured at 3 positions in the scan, 1 central position and 2 positions left and right. Results were averaged. All scans were analyzed by an author who was blinded with regard to the treatment of the eyes.

### Measurements of dopamine and DOPAC with HPLC-ED

Because dopamine levels cycle diurnally [31], preparation times in all experimental groups were between 12:30 and 13:30. Animals were euthanized and after about 2 min, the eyes were enucleated. Working experience had shown that a short rest period prior to dissection facilitates separation of the different layers. The eyeballs were cut with a razor blade perpendicular to the anterior–posterior axis, approximately 1 mm posterior to the ora serrata. The anterior segment of the eye was discarded. The gelatinous vitreous was removed from the posterior segment, using a pair of forceps. Samples of vitreous were immediately frozen in liquid nitrogen. An 8-mm tissue sample was taken from the posterior eye cup using a biopsy punch and transferred to a petri dish under a dissecting microscope, orientation retina up. Usually the retina was just “swimming” on top and could easily be separated from the pigment epithelium which was peeled off and discarded. The remaining choroid was separated from the sclera by the use of forceps and dissecting needles. Possible small residual RPE cell clusters on the choroid could be carefully brushed off under visual control. Some training was necessary to quickly separate the different fundal layers but it was technically well feasible. Retina and choroid were frozen in liquid nitrogen and all tissues were stored at − 80 °C. All vitreous samples were weighed and homogenized in 750 µL mobile phase (Thermo Fisher Scientific, Sunnyvale, CA, USA) while each retina and choroid was mixed with 500 µL mobile phase. The tissues were then disrupted using 5-mm stainless steel beads and a tissue lyser (TissuesLyser LT, Qiagen, Hilden, Germany) at 50 Hz for 3 min. Then 25 µL of retinal and 50 µL of choroidal homogenate were taken out for later protein determination (Pierce BCA Protein kit; Thermo Scientific, Rockford, IL, USA). The retinal, choroidal, and vitreal homogenates were centrifuged at 14,000 g for 10 min, the supernatant was filtered through a 0.2-µm nylon membrane sample filter (Thermo Fisher Scientific), and 25 µL was directly injected into the HPLC system. Samples were analyzed for biogenic amine content by ion-pair reverse phase HPLC with coulometric detection (Ultimate 3000 LC with electrochemical detection ECD3000RS, Thermo Fischer Scientific, California, USA). A hypersil C18 column was used (150 × 3 mm, 3 µm), and the system was run with a test mobile phase containing 10% acetonitrile and 1% phosphate buffer (Thermo Fischer Scientific, California, USA) at a flow rate of 0.4 mL/min. The potential of the first channel was set to + 370 mV, and the second channel to − 200 mV. Dopamine and one of its metabolites DOPAC (3,4-Dihydroxyphenylacetic acid) concentrations were determined by comparing peak areas of the samples with those of standards using Chromeleon 7 chromatography data system software. Dopamine metabolites in standards were determined with a high correlation linearity (r^2^ = 0.98) and good reproducibility in retention time (0.03%); the limit of detection on column was smaller than 1 pg. In retina and choroid, biogenic amine content was determined as nanogram per milligram protein (ng/mg protein), whereas in the vitreous, DOPAC and dopamine amounts were determined relative to wet weight (ng/0.1 g wet weight).

### Exposure to 1 Hz, 10 Hz, and 400 Hz flickering light conditions

Chickens were placed into a cage that was covered with an array of 5 equally spaced strips with 6 white LEDs each (Thomsen white LED 30 W, 5 mm, Conrad Electronics, Hirschau, Germany) on top. The cage was set up in a dark room so that, except for the flickering light, no light from external sources could enter. Flickering light was generated by a function generator (JDS6600-LITE, Dual-Channel Signal Generator, Conrad Electronics), combined with a 20 W audio amplifier. The function generator was set to provide a square wave signal with 20% duty cycle and 97.7% modulation at either 1, 10, or 400 Hz, generating an average illuminance in the cage of about 20 lx. Frequencies were selected based on findings that low frequencies induce myopia in a variety of animal models (i.e., Di et al. [32], guinea pig) while around 10 Hz was found to inhibit deprivation myopia [33] and to stimulate retinal dopamine release [34, 35]. At the highest frequency of 400 Hz, flicker was not detectable by the chickens (electrophysiological and behavioral flicker fusion frequency around 100 Hz, Lisney et al. [36, 37]). This frequency was used to provide a spectrally matched and equiluminant reference lighting for the flickering light stimuli. Choroidal thickness was measured using OCT, before and during exposure to flickering light, every 2–3 h over the whole day. All measurements took place in the dark room. Choroidal thickness data of chickens kept in a standard cage at laboratory lighting (470 lx) were collected for comparison.

### Tissue preparation and immunohistochemistry

Animals were sacrificed by an overdose of ether. Eyes were immediately enucleated and cut with a razor blade in the equatorial plane, approximately 1 mm posterior to the ora serrata. The anterior segment of the eye was discarded and the gel vitreous removed. Tissues were fixed, cryo-sectioned, and immune-labeled as described in detail elsewhere [38]. In short, fixation occurred by immersion in 4% paraformaldehyde plus 3% sucrose in 0.1 M phosphate buffer (pH 7.4) for 30 min at room temperature. Fixed samples were washed three times (10 min each time) in phosphate-buffered saline (PBS; pH 7.4) and cryoprotected in PBS plus 30% sucrose overnight at 4 °C. They were then soaked in embedding medium (Tissue Freezing Medium; Jung, Nussloch, Germany) for 5 min before freezing. Vertical Sects. 12 µm thick were cut and thaw mounted onto silane-coated glass slides. Sections from contralateral control and treated eyes from the same animal were placed consecutively on the same slide to ensure equal exposure. Sections were washed three times in PBS (10 min each time), incubated with blocking buffer (PBS plus 0.3% Triton X-100 (PBST; Sigma-Aldrich, Taufkirchen, Germany) plus 10% normal goat serum [Sigma-Aldrich]), covered with primary antibody solution (200 µL of antiserum in PBST plus 20% normal goat serum), and incubated for about 20 h at room temperature in the dark. Slides were washed three times in PBS (10 min each time), covered with secondary antibody solution (1:500 Alexa 488 goat anti-mouse IgG (Invitrogen, Molecular Probes, Leiden, The Netherlands) or 1:1000 Alexa 568 goat anti-rabbit IgG) and incubated for 2 h at room temperature in the dark. Samples were washed three times in PBS (10 min each time) and mounted under coverslips in H-1000 Vectashield (Vector laboratories, Inc. Burlingame, CA, USA) for observation with a fluorescence microscope. In double-labeling experiments, sections were first incubated with a mixture of two primary antibodies (see below), and second with a mixture of the corresponding secondary antibodies; the respective working dilutions of the antibodies remained unchanged. Application of the respective primary second antibody did not change the labeling of the respective primary first antibody when solely applied. We took this as evidence that no significant cross-reaction between the two primary antibodies existed. Note that different retinal sections from the same animal could be stained for different epitopes. The primary antibodies used in the present study have been already well characterized in the chicken in the past and we evaluated their specificity by comparison with published examples of labeling to their targeted epitopes: anti-alpha2A-ADR and anti-nNOS, e.g., [16]; anti-TH, e.g., [24]; and anti-VEGF, e.g., [28]. Furthermore, specificity for all stainings was assessed by omission of the respective primary antibodies. Primary antibodies and their working dilutions are listed in Table 1.

**Table 1:** List of antibodies, their origins, and working dilutions

Antigen	Antibody	Origin	Working dilution
α_2A_-adrenergic receptor	Anti-α_2A_-adrenergic receptor rabbit polyclonal, ab 92,650	Abcam, Cambridge, UK	1:100
Nitric oxide synthase (NOS)	Anti-NOS, rabbit polyclonal, ALX-210–529	ENZO Life Sciences GmbH, Loerrach, Germany	1:200
Tyrosine hydroxylase(TH)	Anti-TH, mouse monoclonal, 22,941	Immunostar Hudson WI, USA	1:1000
Vascular endothelial growth factor A (VEGF)	Anti-VEGF, mouse monoclonal, orb191500	Biorbyt Ltd Cambridge, Cambridgeshire, UK	1:200

### Experimental groups and treatments

Descriptions of experiments 1–3, number of animals, and treatment protocols are listed in Tables 2, 3, and 4.

**Table 2:** Experiment 1: OCT and HPLC measurements after intravitreal atropine and/or spiperone injections

	Chickens	Intravitreal injection	Treatment
Experiment 1A	7	At 10:00, a single intravitreal atropine injection into one eye, saline vehicle into the other	OCT measurements 1 h before (baseline) and 2, 4, 6, and 7 h after injections; final measurement 24 h after baseline measurements
	12 (also used in Exp 1B)	At 10:00, a single intravitreal combined atropine/spiperone injection, vehicle only into the other	OCT measurements 1 h before (baseline) and 2, 4, 6, and 7 h after injections; final measurement 24 h after baseline measurements
Experiment 1B	7	At 10:00, a single intravitreal atropine injection into one eye, saline vehicle into the other	After about 2 h, animals were euthanized and prepared for HPLC-ED to determine vitreal, retinal, and choroidal levels of dopamine and DOPAC. At this time point, dopamine release was highest, as found in a previous study [16] (same for all HPLC experiments, see below)
	11	At 10:00, a single intravitreal combined atropine/spiperone injection, vehicle only into the other	After about 2 h, animals were euthanized and prepared for HPLC-ED to determine vitreal, retinal, and choroidal levels of dopamine and DOPAC
Experiment 1C	12	At 10:00, a single intravitreal spiperone injection into one eye, vehicle only into the other	OCT measurements 1 h before (baseline) and 2, 3, 5, 7, and 9 h after injection

**Table 3:** Experiment 2: OCT and HPLC measurements after exposure to different flickering light conditions

	Chickens	Flickering light condition	Treatment
2A	8	Broadband white room light (470 lx)	OCT measurements at 9:00 (baseline) and 2, 3, 5, 7, and 8 h after baseline measurements
2B	6	Broadband white room light (470 lx)	After exposure of about 2 h, animals were euthanized and prepared for HPLC-ED to determine vitreal, retinal, and choroidal levels of dopamine and DOPAC
2C	6/6/6 (also used in Exp 2D)	Flickering light (duty cycle 0.2, average luminance 20 lx) 1 Hz, 10 Hz, 400 Hz	OCT measurements at 9:00 (baseline), then transfer to either 1 Hz, 10 Hz, or 400 Hz flickering light, continued OCT measurements 2, 3, 5, 7, and 8 h after baseline measurements
2D	6/6/6	Flickering light (duty cycle 0.2, average luminance 20 lx) 1 Hz/10 Hz/400 Hz	After exposure of about 2 h in either 1 Hz, 10 Hz, or 400 Hz flickering light, animals were euthanized and prepared for HPLC-ED to determine vitreal, retinal, and choroidal levels of dopamine and DOPAC

**Table 4:** Experiment 3: Immunohistochemistry

	Chickens	Lighting	Treatment
3	6	Broadband white room light (470 lx)	None, naïve controls Localization of tyrosine hydroxylase (TH) in choroid. Double-labeling of TH with neuronal nitric oxide synthase (nNOS), vascular endothelial growth factor (VEGF), and alpha2A adrenoreceptors (α2A-ADR)

### Statistics

Statistical analyses were done using commercial software “JMP 13” (SAS Institute, Cary, NC, USA). Data are shown as the mean ± standard deviation. A mixed model repeated measure ANOVA (with time as the repeated measures and group as between subject factor) was used for comparisons of mean choroidal thickness among different treatment groups, followed by a Tukey–Kramer honestly significant difference (HSD) test for post hoc analysis. Two-tailed unpaired Student’s *t*-test was used to compare dopamine and DOPAC contents in different treatment groups at the same time point. Two-tailed paired Student’s *t*-test was used to compare metabolite content in drug-injected and contralateral vehicle-injected eyes in the same animal. Bonferroni corrections were applied in the *t*-test during post hoc analyses. Adjusted p values of *p* < 0.05 were considered significant.

## Results

### Experiment 1—effects of a dopamine antagonist on atropine-induced choroidal thickening

**Fig. 1:**
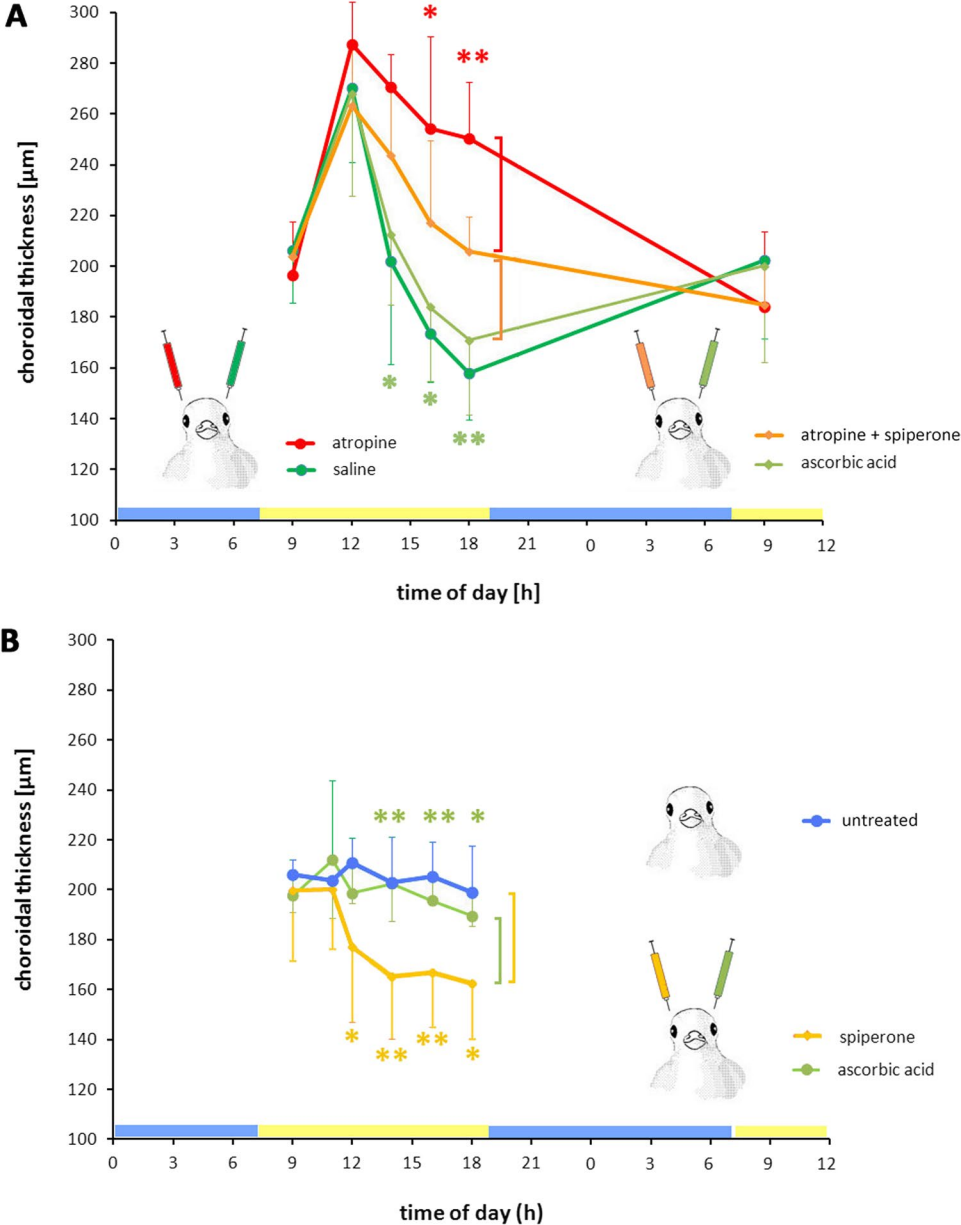
Effects of a D2 antagonist, spiperone, on choroidal thickness over the day. **A** Intravitreal atropine alone (red) caused prominent choroidal thickening for a few hours which was reduced by co-application of spiperone (orange). If atropine was applied to one eye, also the saline- (dark green) or ascorbic acid– (light green) injected fellow eye developed a thicker choroid, although with faster recovery to baseline (*n* = 7 chickens, one eye injected with atropine, the other with saline; *n* = 12 chickens, one eye combined atropine + spiperone injection, the other with vehicle). All eyes recovered to baseline during the following night. **B** Untreated eyes (blue, *n* = 8 untreated chickens) or eyes injected with ascorbic acid (light green) showed no choroidal thickness changes over the day. However, spiperone caused long-lasting choroidal thinning (yellow) (*n* = 12 chickens, one eye injected with spiperone, the other with ascorbic acid). The blue and yellow bars in the bottom denote diurnal dark and light cycles. Colors of brackets denote which data were compared, as denoted by asterisks of the same color. Two-way repeated measures ANOVA with Tukey–Kramer post hoc test; * *p* < 0.05, ** *p* < 0.01

We had previously found that intravitreal atropine causes prominent, but transient, choroidal thickening in the chicken eye. The effect was also observed in the contralateral eye where it was more transient [11]. Time courses and magnitudes of choroidal thickness changes in atropine-injected eyes and their saline-injected fellow eyes closely matched observations from our previous study [11]. To test whether the effect involves dopamine signaling, a dopamine D2 antagonist, spiperone, was co-applied with atropine (Fig. 1).

Spiperone reduced atropine-induced choroidal thickening (Fig. 1A): spiperone/atropine-injected eyes: mean choroidal thickness 217.18 ± 32.48 µm (16:00) and 205.97 ± 13.44 µm (18:00), versus atropine-injected eyes 254.22 ± 36.27 µm (16:00) and 184.12 ± 29.52 µm (18:00); *p* < 0.05 and p < 0.01, respectively. Nevertheless, the choroid remained still thicker than in ascorbic acid–injected or saline-injected eyes (spiperone/atropine-injected eyes: mean choroidal thickness 243.54 ± 25.85 µm (14:00), 217.18 ± 32.48 µm (16:00), and 205.97 ± 13.44 µm (18:00), versus ascorbic acid–injected eye 212.44 ± 27.5 µm (14:00), 183.88 ± 29.07 µm (16:00), and 170.93 ± 29.39 µm (18:00); *p* < 0.05, *p* < 0.05, and *p* < 0.01, respectively). Furthermore, the effects of atropine were clearly visible also in the fellow eyes, as described before [11] but the effect of spiperone was not. An intravitreal spiperone injection in the morning made the choroid thinner over the whole day, compared to untreated or ascorbic acid–injected eyes (Fig. 1B), with no signs of recovery on this day (spiperone-injected eyes: mean choroidal thickness 165.19 ± 24.91 µm (14:00), 166.68 ± 21.65 µm (16:00), and 162.38 ± 22.32 µm (18:00), versus ascorbic acid–injected eyes 202.45 ± 18.91 µm (14:00), 195.62 ± 23.57 µm (16:00), and 189.48 ± 28.19 µm (18:00); *p* < 0.01, *p* < 0.01, and *p* < 0.05, respectively).

**Fig. 2:**
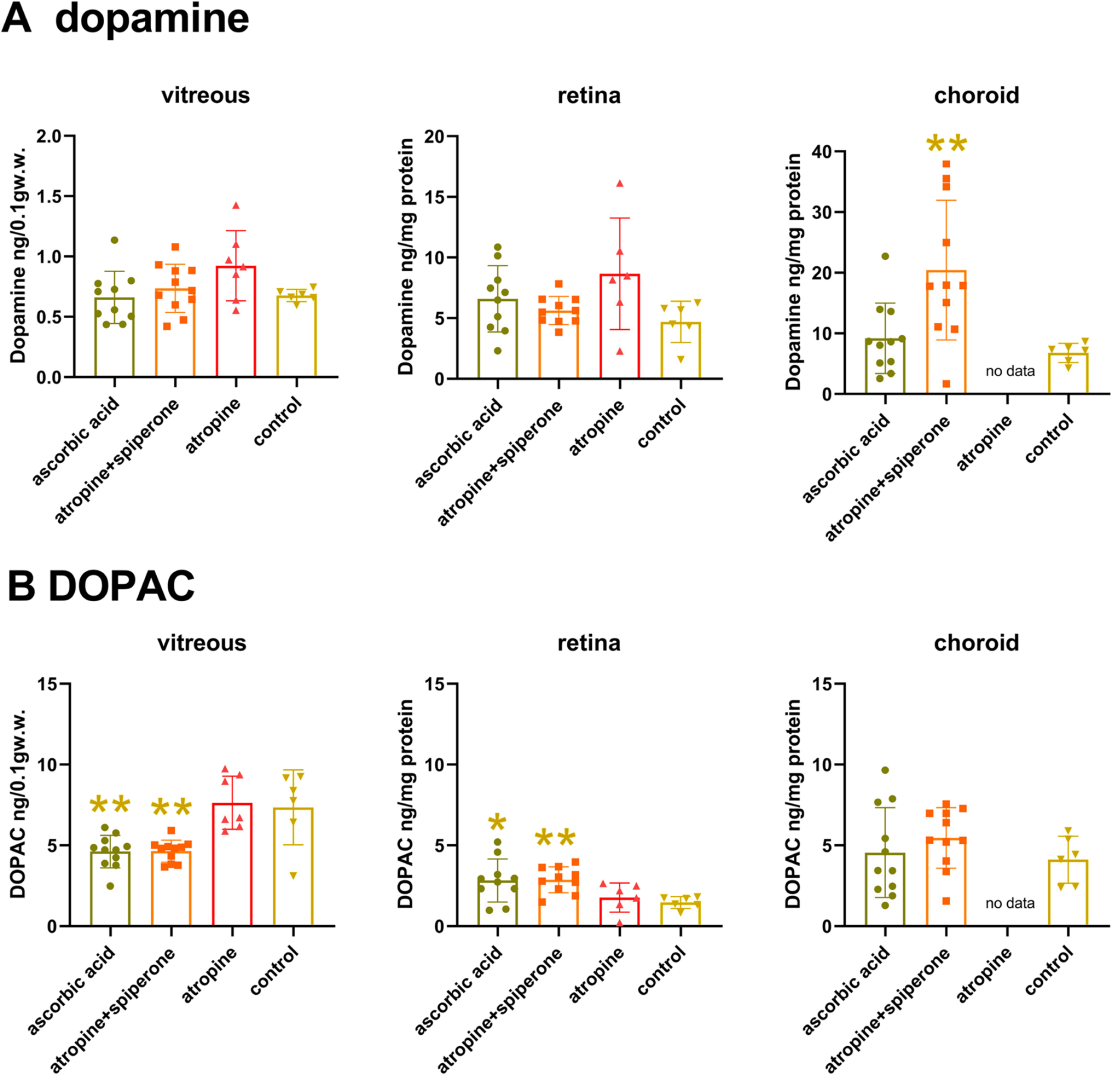
Effects of intravitreal application of vehicle ascorbic acid (green, *n* = 11 eyes), atropine + spiperone (orange, *n* = 11 eyes), atropine (red, *n* = 7 eyes), and no injection (yellow, *n* = 6 eyes) on dopamine and DOPAC content, 2 h after injections. **A** There were no significant effects on dopamine content in vitreous and retina in spiperone + atropine–injected eyes, compared to atropine alone, but dopamine was clearly increased in the choroid in atropine + spiperone–injected eyes. **B** Atropine + spiperone injections reduced DOPAC content in the vitreous, compared to atropine alone, or to untreated eyes with no injection at all or to eyes injected with ascorbic acid (green bar). In the retina, DOPAC was elevated both after ascorbic acid and atropine + spiperone injections, compared to atropine alone and non-injected eyes. Two-tailed unpaired Student’s *t*-tests; *p < 0.05, **p < 0.01. Colors of asterisks match the color of the data column for comparison

In control eyes (Fig. 2, yellow bars), dopamine levels in the choroid (6.7 ± 1.6 ng/mg protein) and the retina (5.2 ± 2.1 ng/mg protein) were similar (Fig. 2A) but there was clearly less dopamine in the vitreous. Intravitreal injection of 250 µg atropine (red bar in Fig. 2A) showed a trend to increase retinal (8.7 ± 4.5 ng/mg protein) and vitreal (0.9 ± 0.3 ng/0.1gw.w.) dopamine content which was however not significant. Atropine combined with spiperone (Fig. 2A, orange bars) had no effect on dopamine (retina: 5.63 ± 1.15 ng/mg protein; vitreous: 0.74 ± 0.2 ng/0.1gw.w.), similar to ascorbic acid injections (retina: 6.59 ± 2.73 ng/mg protein; vitreous: 0.77 ± 0.41 ng/0.1gw.w.) (Fig. 2A, green bars). However, it increased dopamine content in the choroid by a factor of about 3 (22.31 ± 10.22 ng/mg protein; *p* < 0.01).

Atropine had no effect on DOPAC in the retina (1.77 ± 0.9 ng/mg protein) or vitreous (7.64 ± 1.65 ng/0.1gw.w.; Fig. 2B, red bar; no data available for the choroid). However, when applied together with spiperone, retinal DOPAC content increased (2.87 ± 0.8 ng/mg protein; *p* < 0.01), compared to the untreated control. The effect must, however, be unspecific since similar changes were observed with ascorbic acid (2.83 ± 1.33 ng/mg protein). In vitreous, combined application of atropine and spiperone reduced DOPAC content (4.64 ± 0.68 ng/0.1gw.w.), compared to atropine alone (7.64 ± 1.65 ng/0.1 gw.w.; *p* < 0.0001). Again, a similar effect was observed for ascorbic acid (4.61 ± 1.01 ng/0.1 gw.w.) versus atropine (7.64 ± 1.65 ng/0.1 gw.w.; *p* < 0.001).

### Experiment 2—effects of flickering light on choroidal thickness and dopamine

**Fig. 3:**
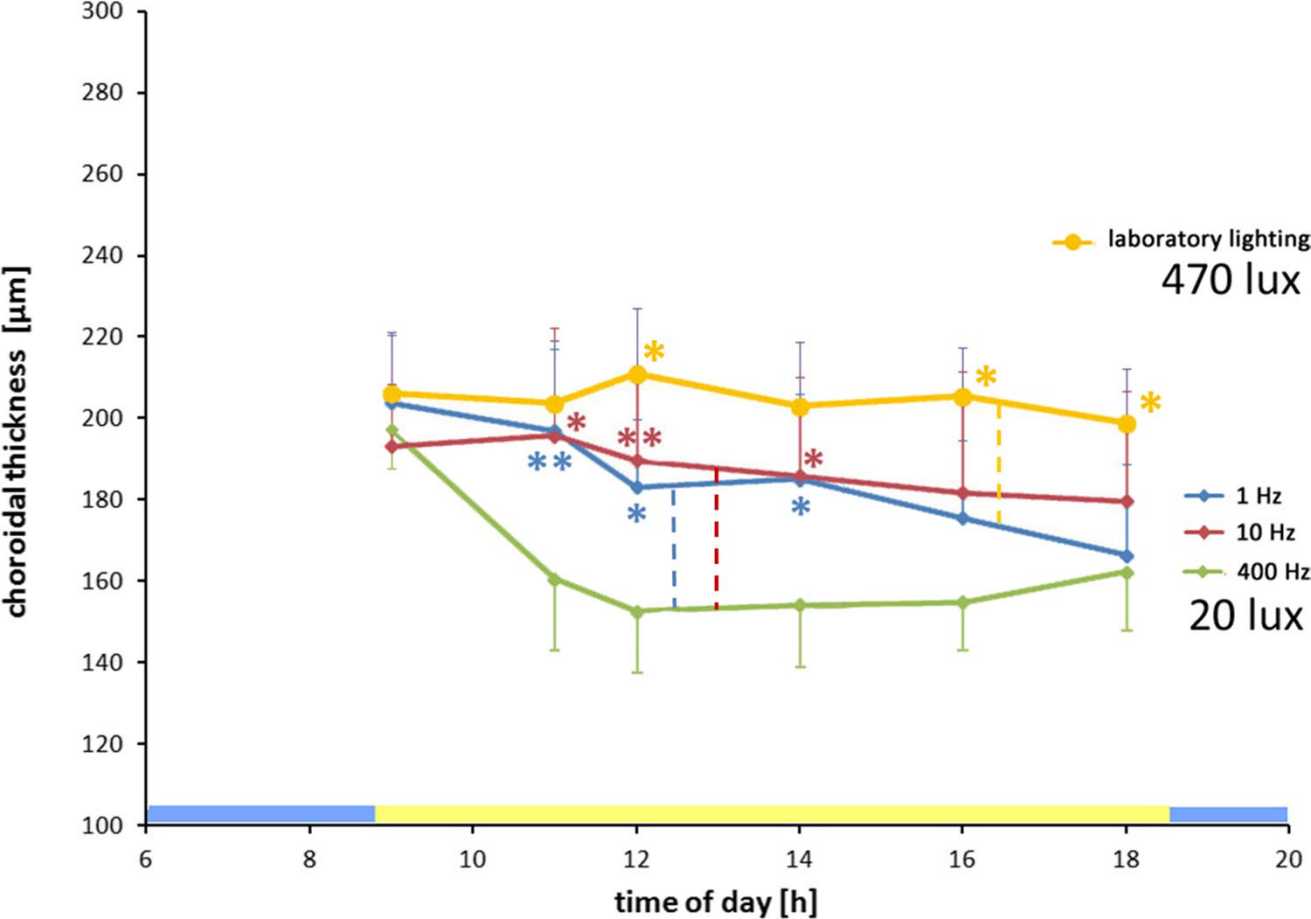
Effects of flickering light emitted from white LEDs on choroidal thickness over the day. With 400 Hz flicker at 20 lx (green; seen as continuous light by the chicks), the choroid became rapidly thinner over the first few hours but then remained constant. When 20 lx light was flickered, the choroid became thicker both at 1 Hz (blue) and 10 Hz (red) but did not reach the thickness that was developed under brighter laboratory lighting (470 lx, yellow line) (*n* = 6 chickens at 1 Hz, 10 Hz, and 400 Hz respectively; *n* = 8 chickens under laboratory lighting). The blue and yellow bars in the bottom denote diurnal dark and light cycles. Colors of the asteriks match the colors of the interrupted lines denoting which data were compared. Two-way repeated measures ANOVA with Tukey–Kramer post hoc test;*p < 0.05, **p < 0.01

To test further how dopamine release from the retina might be involved in the control of choroidal thickness, we used another way to stimulate dopamine. It is known since 1971 [39] that dopamine release is stimulated by flickering light [39–43]. Therefore, chickens were exposed to flickering light for 1 day with 1 Hz, 10 Hz, and 400 Hz and a 20% duty cycle. Light was provided by an array of white LEDs and, for technical reasons, only an average illuminance of 20 lx was possible for the used flicker parameters. Laboratory lighting which was about 24 times brighter (470 lx) was also tested. Under flickering light at 1 and 10 Hz, the choroid remained thicker than under 400 Hz even though all had the same average illuminance (20 lx, green, Fig. 3): mean choroidal thickness (µm) 1 Hz 11:00 196.95 ± 20.25, 12:00 183.14 ± 18.83, and 14:00 185.04 ± 20.9 and 10 Hz 11:00 195.75 ± 26.51, 12:00 189.75 ± 23.29, and 14:00 185.86 ± 24.28 versus 400 Hz 11:00 160.48 ± 11.71, 12:00 152.59 ± 10.01, and 14:00 154.16 ± 13.76; 11:00 *p* < 0.01 and *p* < 0.05; 12:00 *p* < 0.05 and *p* < 0.01; and 14:00 *p* < 0.05 and *p* < 0.05, respectively. No differences were found in choroidal thickness under 1 and 10 Hz flickering light (Fig. 3). However, the choroid remained thicker under laboratory lighting (470 lx) than under flickering light at 20 lx and 1 Hz (*p* < 0.05 at 12:00, 16:00, and 18:00).

**Fig. 4:**
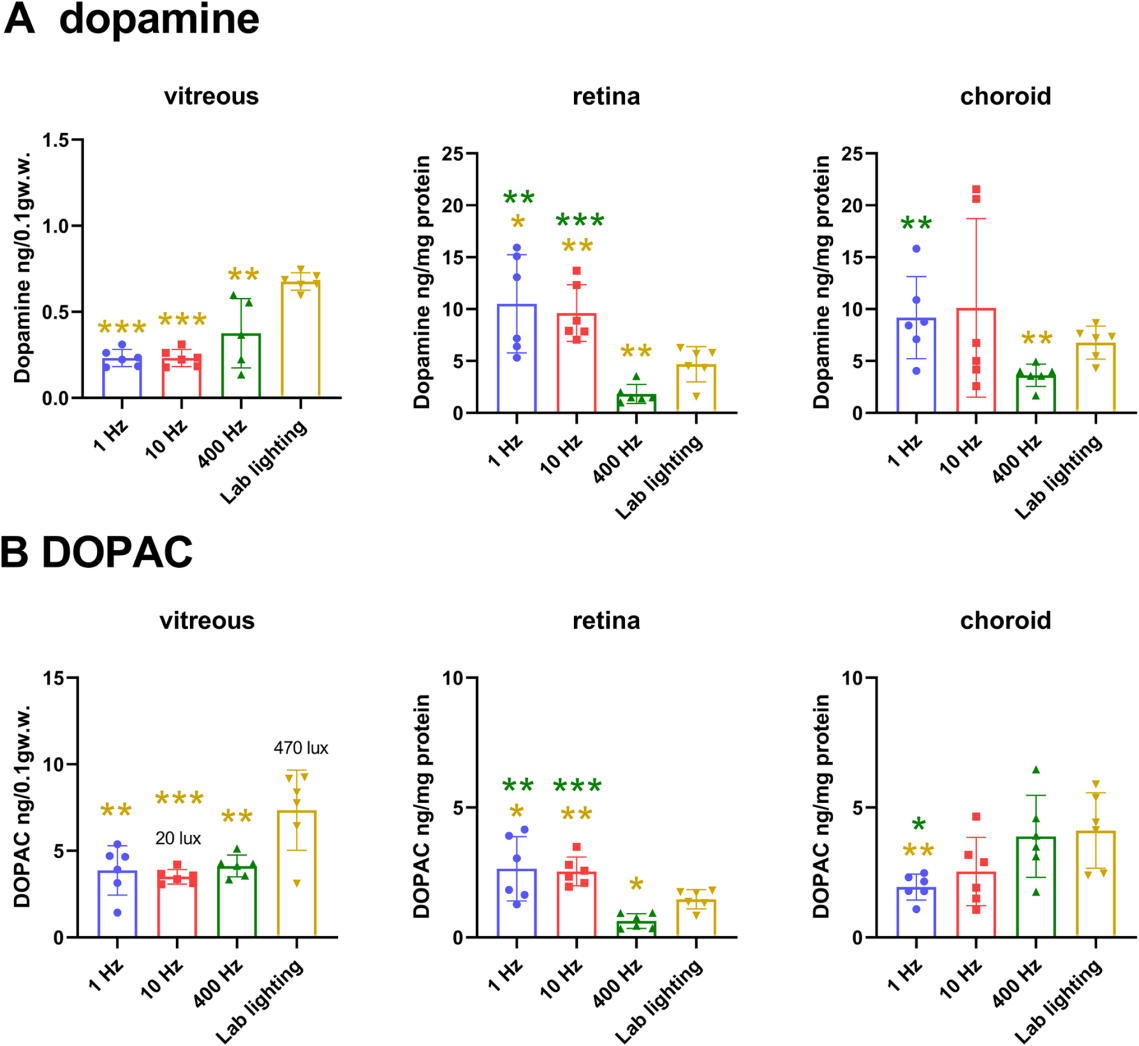
Vitreal, retinal, and choroidal dopamine and DOPAC levels in chickens exposed to flickering light at 1, 10, or 400 Hz at 20 lx (*n* = 6 in each case), and under laboratory lighting at 470 lx (*n* = 6). Flickering light, both at 1 and 10 Hz, increased retinal dopamine and DOPAC content more than increasing illuminance from 20 to 470 lx (middle) while 400 Hz flickering light at 20 lx caused a significant decrease in retinal and vitreal DA and DOPAC levels (middle). Flickering light at 20 lx also reduced vitreal dopamine and DOPAC content, compared to laboratory lighting at 470 lx (left). One hertz flickering light also reduced choroidal dopamine and DOPAC content (right). Two-tailed unpaired Student’s *t*-test; *p < 0.05, **p < 0.01, ***p < 0.001. Colors of asterisks match the color of the data column for comparison

HPLC measurements confirmed that flickering light stimulated dopamine production in the retina. Both 1 and 10 Hz flickering light changed dopamine and DOPAC in a similar way in all 3 measured tissue layers, vitreous, retina, and choroid (Fig. 4A, red and blue bars). Retinal dopamine content under flickering light (1 Hz: 10.50 ± 1.72; 10 Hz: 9.61 ± 2.73 ng/mg protein) was twice as high as under continuous laboratory lighting (4.69 ± 1.70 ng/mg protein) (yellow bar) (*p* < 0.05 and *p* < 0.01, respectively) even though the illuminance was lower by a factor of 17.5 (green bar). This confirms that flickering light stimulates retinal dopamine more than just elevating illuminances. The pattern was similar in the choroid (Fig. 4A, right) although a significant increase was only achieved relative to the 400 Hz at 20 lx light (1 Hz: 9.18 ± 3.96 vs. 400 Hz: 4.4 ± 1.72 ng/mg protein, *p* < 0.01; compared to laboratory lighting: 6.77 ± 1.59 ng/mg protein, n.s.). Dopamine levels in the retina changed in the same direction as the levels of DOPAC in vitreous and retina (Fig. 4B). Vitreal DOPAC and dopamine were highest under laboratory lighting (DOPAC: 6.80 ± 1.44 ng/0.1 gw.w.; dopamine: 0.68 ± 0.05 ng/mg protein). However, in the choroid, DOPAC was significantly reduced under 1 Hz (1.98 ± 0.50 ng/0.1gw.w.) flickering light, compared to 400 Hz (3.89 ± 1.58 ng/0.1 gw.w.) at 20 lx (*p* < 0.05) and laboratory light at 470 Hz (4.11 ± 1.45 ng/0.1 gw.w.; *p* < 0.01). It can be summarized that 1 and 10 Hz flickering light exposure for 2 h increased dopamine in retina and choroid, compared to 400 Hz.

### Correlation analyses: choroidal thickness versus dopamine and DOPAC levels

**Fig. 5:**
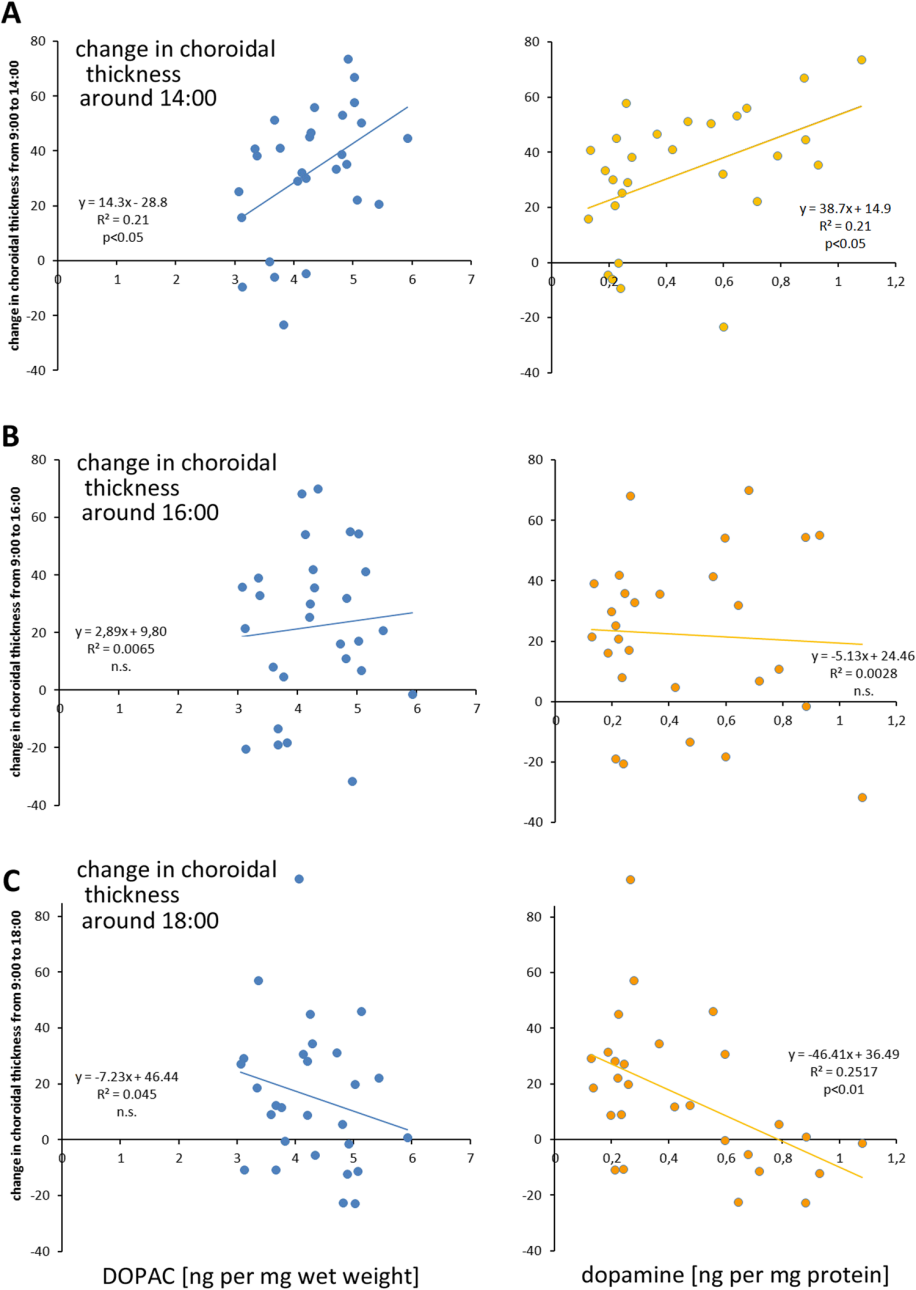
Correlation analyses of choroidal thickness changes measured at 3 time points with DOPAC and dopamine levels in vitreous as measured around noon. While choroidal thickness was positively correlated with DOPAC and dopamine levels in the vitreous at 14:00, the correlation inverted during the following four hours

To further evaluate potential links between dopaminergic transmission and choroidal thickness, changes in choroidal thickness at 3 time points (14:00, 16:00, and 18:00) were correlated with the changes in DOPAC and dopamine as measured around noon. It is likely that a correlation that persists across different experiments (atropin/spiperone treatment and flicker experiments) makes a stronger case that both variables are correlated. Correlation analyses included data from both spiperone and flicker experiments. Choroidal thickness data were collected in the same chickens a day before the HPLC data where it was necessary to sacrifice the animals. On both days, chickens had undergone the same treatments (either atropine + spiperone injection (Table 2: experiments 1A and 1B) or flickering light (Table 3: experiments 2C and 2D)). Significant correlations were found for vitreal DOPAC and dopamine: the higher vitreal DOPAC and dopamine content, the thicker was the choroid around 14:00 (Fig. 5A). The correlation disappeared 2 h later around 16:00 and was inverted around 18:00, reaching significance (*p* < 0.01) for dopamine although a trend was seen also for DOPAC. Results suggest that the initial choroidal thickening rate was associated with an increase in vitreal DOPAC and dopamine which indicates more dopamine release from the retina [26, 44]. Interestingly, changes of DOPAC and dopamine in the choroid itself were not significantly associated with its thickness (data not shown).

The inversion of the correlation around 18:00 could be explained by the time courses of choroidal thickness changes over the day (Figs. 1 and 3), by diurnal variations of DOPAC and dopamine release from the retina (which we did not measure), or by the assumption that the initial change in dopamine triggered only a transient increase in choroidal thickness which returned to baseline over the day. Nevertheless, vitreal dopamine levels had some predictive power for the development of choroidal thickness over the day since the choroid became thinner, the higher the vitreal dopamine levels were at noon (see Fig. 5A versus C).

### Correlation analyses: DOPAC and dopamine in vitreous, retina, and choroid

Different from observations by Megaw et al. [44], vitreal DOPAC and retinal dopamine were not correlated in the current study when all chickens were included (vitreal DOPAC =  − 0.67*retinal dopamine + 10.04, R^2^ = 0.017, *n* = 29, n.s.). There are a number of partially highly significant correlations among DOPAC and dopamine in the three studied tissues (vitreous, retina, and choroid). These correlations are unlikely to be random but rather reflect interactions of DOPAC and dopamine in these layers. Significant correlations were found between choroidal DOPAC and vitreal dopamine (y = 4.22x + 2.07, R^2^ = 0.346, *p* < 0.01), choroidal DOPAC and retinal dopamine (y =  − 0.22x + 5.54, R^2^ = 0.176, *p* = 0.05), vitreal and retinal dopamine (y =  − 4.72x + 9.33, R^2^ = 0.117, *p* = 0.05), and vitreal and choroidal dopamine (y = 20.20x + 3.81, R^2^ = 0.298, p < 0.01) (not graphically represented as a figure).

**Fig. 6:**
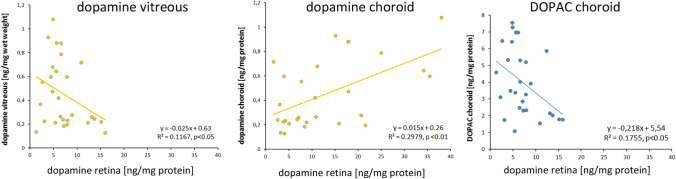
Correlations of retinal dopamine content with dopamine content in vitreous and choroid, and with DOPAC in choroid

Dopamine in the retina was correlated with dopamine in the vitreous, dopamine in the choroid, and DOPAC in the choroid (Fig. 6). While the pattern may look complicated, it can be summarized as that an increase in retinal dopamine is correlated with an increase in choroidal dopamine and a drop in vitreal dopamine and choroidal DOPAC.

### Experiment 3: histological studies in the choroid

**Fig. 7:**
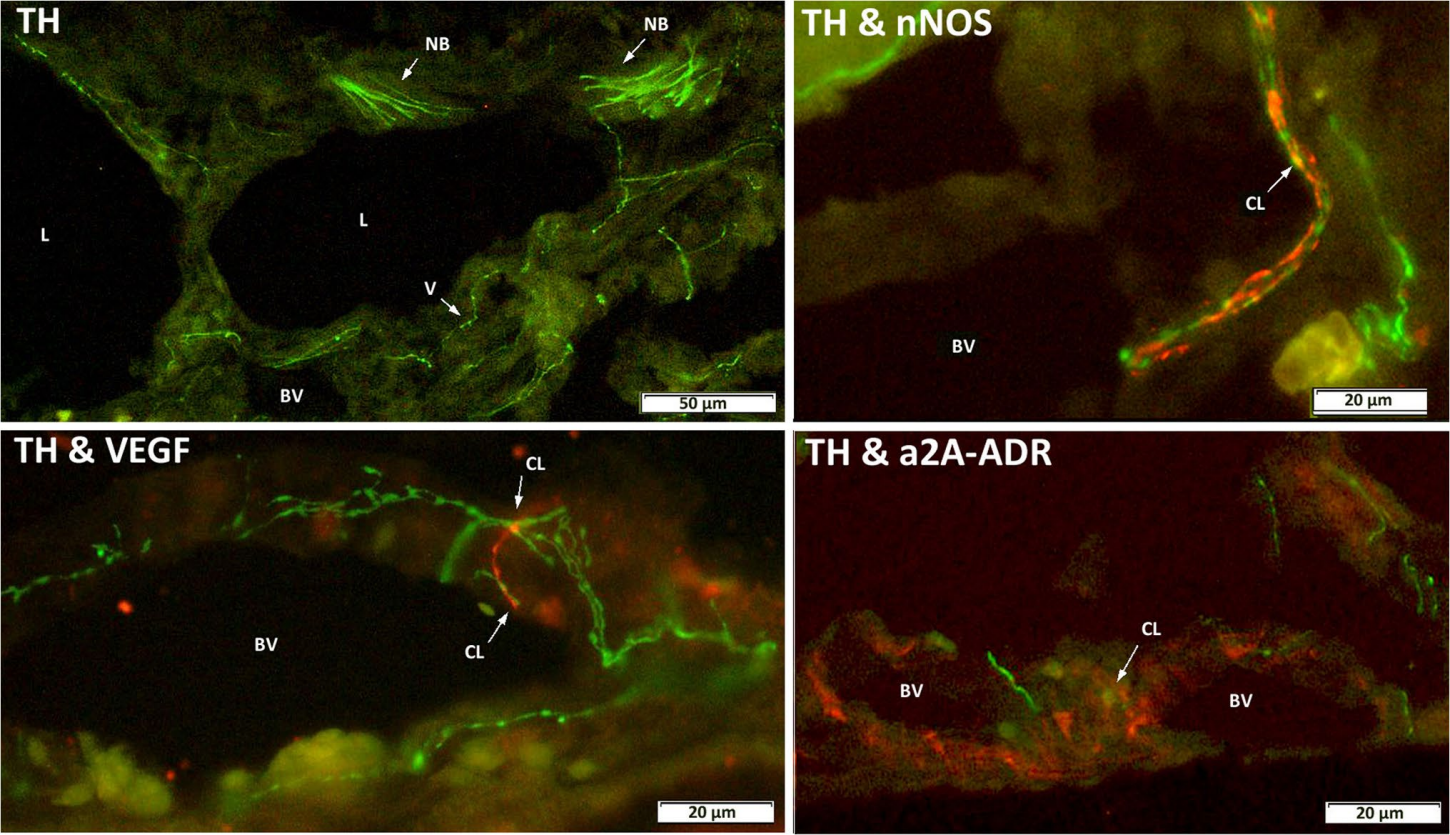
TH immunoreactivity (green) in the chicken choroid. A vertical section shows varicose nerve fibers (V) and nerve fiber bundles (NB) in the entire choroid around lacunae (L) and in the walls of blood vessels (BV). Merged micrographs of double-stained sections with nNOS, VEGF, or a2A-ADR (in red respectively) reveal close contact to TH-positive fibers and/or show colocalizations (yellowish orange) at various sites in choroidal tissue (see white arrows). CL colocalization, BV blood vessel

In the chicken, we localized TH immunoreactivity in varicose nerve fibers and axon bundles which spread over the entire choroid, in the suprachoroid, in choroidal stroma tissue, around lacunae, and in the walls of blood vessels; an overview is shown in Fig. 7, upper left. In double staining experiments, we analyzed possible contacts of TH-positive fibers with nNOS, VEGF, and alpha2a adrenoreceptor immunoreactive cells. NO was previously shown to play a role in ocular growth in the chicken by Nickla et al. [45, 46]. In the current study, we localized many nNOS-positive neurons over the entire choroid which showed close contact to varicose TH immunoreactive nerve fibers, sometimes twined around each other, and/or were co-stained with TH at possible synaptic sites (examples can be seen in Fig. 7, upper right panel).

In an earlier study, we had found in chickens that bevacizumab, an anti-VEGF antibody, suppressed choroidal thickening that normally occurs when eyes recover from induced myopia [28]. We localized VEGF to the walls of choroidal blood vessels and in choroidal nerve fiber bundles. In the current double staining experiments, VEGF-positive fibers show numerous close appositions of TH-immunoreactive varicose nerve fibers; co-stained varicose buttons suggest close nonsynaptic or synaptic contacts (Fig. 7, bottom right). Carr et al. [47] had found in the chicken that atropine may inhibit myopia also by binding to alpha2a adrenergic receptors (alpha2a ADR), and we had previously found that alpha2a ADRs are expressed on dopaminergic retinal neurons [16]. In the current study, we found prominent alpha2a ARD immunoreactivity on the walls of choroidal blood vessels and in choroidal stroma where TH-positive varicose nerve fibers appear in close contact and/or are co-stained with single varicosities (Fig. 7, bottom right).

## Discussion

Using choroidal thickness changes as a measure of potential retinal output signals that control axial eye growth, we found that (1) spiperone, a D2 antagonist, reduced atropine-induced choroidal thickening and also made the choroid thinner without atropine; (2) choroidal thickness remained thicker under continuous bright light than under flickering light at 1 and 10 Hz and became thinnest at 400 Hz (which was far above the flicker fusion frequency); (3) flickering light at 1 and 10 Hz at 20 lx caused higher retinal and vitreal dopamine levels than continuous light at 470 lx even though it was 15 times brighter and also compared to flickering light at 400 Hz; and (4) dopamine and its metabolite DOPAC were also detected in the choroid (although the role of choroidal dopamine is unclear [21]). Both vitreal and choroidal dopamine concentrations were correlated with retinal dopamine concentrations. (5) Choroidal thickening rate was associated with an increase in vitreal DOPAC and dopamine (which indicates more dopamine release from the retina). (6) Tyrosine hydroxylase (TH) was localized in choroidal nerve fibers with apposition to nNOS- and VEGF-positive neurons. Alpha2A adrenoreceptors were located in the walls of choroidal blood vessels, together with TH.

### Finding (1)—inhibition of atropine-induced choroidal thickening by the D2 dopamine antagonist, spiperone

Initially, choroidal thickening was assumed to be independent of dopamine because the choroid still thickened with positive lenses after reserpine or 6-OHDA injections [26] which severely reduce dopamine content (down to about 20%). Later, Nickla et al. [8] stated that D2 receptor agonists that cause growth inhibition also cause transient choroidal thickening. They speculated that the source of dopamine is retinal, and that it acts upstream of the choroidal thickening in response to growth inhibiting visual stimuli. Later, Nickla and Totonelli [48] found that spiperone blocked choroidal thickening that normally occurs when chicks recover from induced myopia which is in line with our findings. Since spiperone blocked the inhibitory effects of bright light on deprivation myopia, Ashby and Schaeffel [23] concluded that myopia inhibition is mediated by dopamine. Nickla et al. [13] proposed that dopamine stimulates nitric oxide (NO), a retinal light signal like dopamine. NO is (partially) mediating choroidal thickening because nitric oxide synthase inhibitors prevent the choroidal thickening response to myopic defocus and disinhibit ocular growth [45, 49]. NO is also stimulated by flickering light [50] and, in fact, we found in the current study that the choroid thickenend in flickering light at 1 and 10 Hz, compared to visually continuous light at 400 Hz. Carr and Stell [51] found that intraocular NO inhibits myopia dose-dependently and is required for inhibition of myopia by atropine. In summary, there is evidence that atropine can thicken the choroid by stimulating NO and dopamine which may both be necessary. However, it is clear that also there is muscarinic control of choroidal thickness since muscarinic agonists thinned the choroid in chicks [46].

Thomson et al. [22] recently excluded a role of dopamine during myopia inhibition by atropine for two reasons. They found that coadministration of the dopamine D2-like receptor antagonist spiperone did not inhibit the protective effects of 0.15 nmol of atropine against deprivation myopia, and that nicotinergic agents can inhibit myopia without affecting dopamine levels. However, their atropine doses were very low (0.1 µg), a dose which had no effect on myopia development in an earlier study by Diether et al. [21]. It is possible that part of the inhibitory effect was due to the intravitreal injection itself, an idea which is in line with the lack of a dose-dependent effect of atropine [22]. Dopamine was measured by mass spectrometry which interestingly showed a DOPAC to dopamine ratio in the vitreous of 150 to 300 while the ratio was about 10 in our HPLC measurements, or even lower in other studies [44, 52]. Another difference was that Thomson et al. [22] measured dopamine 1 h after injection while we measured after about 2 h.

Tian et al. [52] had stated that “The retinal DOPAC/dopamine ratio, indicating the metabolic efficiency of dopamine, is correlated with ocular growth.” Therefore, we also studied whether the DOPAC to dopamine ratios might achieve better correlations with choroidal thickness changes. In line with findings by Tian et al. in guinea pig [52], we also found in the chicken that the retinal DOPAC to dopamine ratios were significantly correlated with the thickness of the choroid (choroidal thickness =  − 15.9 * DOPAC/dopamine ± 187.6, R^2^ = 0.2135, *p* = 0.02). Choroidal DOPAC to dopamine ratios were not correlated with choroidal thickness. Interestingly, the DOPAC/DA ratio was much higher in the choroid (13.17 ± 7.02), compared to retina (6.11 ± 3.80; *p* = 2.86*10 − 5).

### Findings (2) and (3)—link between dopamine levels and choroidal thickness under flickering light and at two different illuminances

It has been found before that flickering light stimulates dopamine release from the chicken retina (i.e., [34, 53, 54]). However, associated changes in choroidal thickness have not been measured before. While we found that the choroid thickened with 1 and 10 Hz flickering light, compared to 400 Hz, the choroid was thickest at the higher illuminances of 470 lx. Lan et al. [27] had previously found that the choroid becomes thicker in bright light. Also, vitreal DOPAC was highest at 470 lx. However, dopamine content in the retina was more elevated in dim flickering light compared to continuous bright laboratory light. It would be informative to measure time courses of dopamine release and of choroidal thickening to fully understand these observations. Also, dopamine release may not always correlate with myopia inhibition. In guinea pigs, it was found that myopia induced by (low frequency) flickering light was associated with increased rather than decreased dopamine release [55].

### Finding (4)—evidence for a role of dopamine also in the choroid

Our HPLC measurements in choroidal tissue clearly showed that both dopamine and DOPAC were present in the choroid. Furthermore, both vitreal and choroidal dopamine concentrations were correlated with retinal dopamine concentrations (*p* = 0.02), suggesting that changes of dopamine in the choroid may have a function. However, as supported by finding 6 below, it is unlikely that retinal dopamine can diffuse through the RPE [26]. Brown et al. [18] state that “since there is no evidence of DA receptor expression in the choroid or sclera, it is unlikely that retinal DA has direct effects on these structures during myopia development. Therefore, retinal DA likely acts only in the retina and RPE. Additional research is necessary to elucidate signaling occurring at the RPE and how it may propagate and influence the choroid.”

### Finding (5)—elevated vitreal DOPAC and dopamine correlated with choroidal thickening

While retinal and choroidal dopamine levels were correlated if data from all experiments of the current study are pooled, only vitreal and retinal dopamine levels are linked by diffusion of dopamine [26]. As described above, choroidal dopamine is most likely controlled by secondary messengers that are released on the choroidal side from the retinal pigment epithelium (RPE) and all five dopamine receptor types have been found on the retinal side of the RPE. Nevertheless, the observation that retinal and choriodal dopamine levels were correlated may indicate that dopamine plays also a role in the choroidal thickness changes although dopamine receptors have not yet been localized in choroidal tissue [18].

### Finding (6)—immunohistochemical localization of the key enzyme for dopamine synthesis, TH, in the choroid

Schroedl et al. [56] had identified TH-positive fibers in the choroid of the duck. In double staining experiments, we analyzed possible contacts of TH-positive fibers with nNOS, VEGF, and alpha2a adrenoreceptor immunoreactive cells. NO was shown to play a role in ocular growth in the chicken by Nickla et al. [45, 46]. Nickla et al. [49] also showed that transient choroidal thickening in response to myopic defocus in chicken could be blocked by the nitric oxide synthase inhibitor l-NAME. Also, Carr and Stell [51] found that NO played a role in choroidal thickness regulation since NOS inhibitors blocked the suppressive effect of atropine on form deprivation myopia. NOS-positive immunoreactivity in axon bundles and innervation to vascular smooth muscle in the chicken choroid were already found by Fischer and Stell [57]. Schroedl et al. [56, 58] identified intrinsic choroidal neurons positive for nNOS in the duck and found that they were in contact with TH-positive choroidal nerves. In the current study, we localized many nNOS-positive neurons over the entire choroid which showed close contact to varicose TH immunoreactive nerve fibers.

In an earlier study, we had found in chickens that bevacizumab, an anti-VEGF antibody, suppressed choroidal thickening that normally occurs when eyes recover from induced myopia [28] and localized VEGF to the walls of choroidal blood vessels and in choroidal nerve fiber bundles. In the current double staining experiments, VEGF-positive fibers show numerous close appositions of TH-immunoreactive varicose nerve fibers. Carr et al. [47] had found in the chicken that atropine may inhibit myopia also by binding to alpha2a adrenergic receptors (alpha2a ADR), and we had previously found that alpha2a ADRs are expressed on dopaminergic retinal neurons [16]. In mammals (e.g., [59, 60]), choroidal vasoconstriction caused by direct administration of noradrenaline or by activation of sympathetic nerves to the choroid is mediated by alpha-adrenergic receptors on blood vessels. In the current study, we found prominent alpha2a ARD immunoreactivity on the walls of choroidal blood vessels and in choroidal stroma together with TH-positive nerve fibers.

Finding 6 suggests that dopamine is also produced in the choroid but its role has not been described. There are a number of agents controlling blood flow and choroidal thickness [61]. Studies support the hypothesis that choroidal vasoconstriction is mediated by alpha-adrenergic receptors on blood vessels (for example [60, 62, 63]). Consistent with a role of alpha-adrenergic receptors on choroidal vessels in choroidal blood flow control, Kiel and Lovell [59] reported that alpha-adrenoreceptor blockade increased choroidal blood flow in rabbits. nNos-positive intrinsic choroidal neurons in birds give rise to processes that contact choroidal blood vessels; they give output to the smooth muscle of choroidal blood vessels [64, 65]. It was found that nNOS and VIP have a vasodilalatory influence on choroidal vessels. Reiner et al. [61] also stated that intrinsic choroidal neurons are also of interest for the role they may play in modulating the choroidal thickening that serves to reposition the retina to align it with the image plane [8]. There is innervation of intrinsic choroidal neurons to the extravascular smooth muscle common to the choroid in birds but also seen in primates [66, 67]. The smooth muscle is thought to play a role in the filling or emptying of intrachoroidal lacunae that causes choroidal thickening or thinning, respectively [8, 68]. The thinning is accompanied by reduced choroidal blood flow and the thickening by increased choroidal blood flow [69–71]. As Reiner et al. [61] already suggested, it may be that activation of the intrinsic choroidal neurons drives both the flow increase and the choroidal thickening, and their inhibition the opposite.

In the current study, we have localized several of the agents that were previously proposed to be involved in the control of choroidal blood flow and choroidal thickness. They were colocalized with TH, the rate-limiting enzyme of dopamine. We have found close contacts of TH-positive nerve fibers with nNOS- and VEGF-positive nerve fibers, as well as contacts between TH-positive fibers with alpha2A adrenoreceptors in the walls of the blood vessels.

## Conclusions

Our study is in line with the hypothesis that atropine exerts an effect on choroidal thickness via dopaminergic transmission. We also provide evidence that a dopamine signal is acting in the choroid although previous studies have shown that it does not reach the choroid by diffusion from retinal sources but rather after relay at the RPE through other messengers at the choroidal side of the RPE. Our results expand the knowledge on signals by which the retina controls the thickness of the choroid and are important for the understanding of myopia development.
